# Editorial: Precision Medicine in Chronic Inflammation

**DOI:** 10.3389/fimmu.2021.770462

**Published:** 2021-09-23

**Authors:** Oliver Distler, Ralf J. Ludwig, Stefan Niemann, Gabriela Riemekasten, Stefan Schreiber

**Affiliations:** ^1^ Department of Rheumatology, University Hospital Zurich, University of Zurich, Zurich, Switzerland; ^2^ Lübeck Institute of Experimental Dermatology and Center for Research on Inflammation of the Skin, University of Lübeck, Zurich, Germany; ^3^ Research Center Borstel, Molecular and Experimental Mycobacteriology, Borstel, Germany; ^4^ German Center for Infection Research (DZIF), Partner Site Hamburg-Lübeck-Borstel-Riems, Borstel, Germany; ^5^ Department of Rheumatology, University of Lübeck, Lübeck, Germany; ^6^ Department of Medicine I, University Hospital Schleswig-Holstein, Kiel, Germany

**Keywords:** Precision medicine, autoimmunity, tuberculosis, inflammation, cardiovascular events, nutrition, imaging, patient reported outcomes

The principle of precision medicine is most established in oncology; e.g. choice of particular treatment based on the presence of certain molecular alterations within the tumors (1). This implementation of precision medicine has significantly improved the prognosis across many malignant diseases (2). Compared to oncology, precision medicine is still in its infancy in chronic inflammatory diseases – exemplified for pemphigus and pemphigoid diseases (Bieber et al.) herein. However, implementation of precision medicine for chronic inflammatory diseases, such as chronic infectious diseases, inflammatory bowel disease, inflammatory rheumatic diseases and chronic inflammatory diseases of the skin, is expected to have a significant impact of patient well-being (3). There are three key pillars of precision medicine that will enable its implementation into clinical use: (i) identification of unique disease-associated characteristics in individual patients (ii) personalized experimental models of chronic inflammation, and (iii) implementation of personalized treatments. All of these are highlighted in the articles of the Research Topic *Precision Medicine in Chronic Inflammation*, and are shortly introduced in this Editorial.

## Identification of Unique Disease-Associated Characteristics in Individual Patients

At the forefront of precision medicine is the ability to identify unique characteristics in individual patients allowing to select a tailored treatment. This requires techniques that go well beyond currently implemented diagnostic algorithms. Among methods that allow to differentiate between individual patients are (i) assessment of patient-reported outcomes, (ii) *in vivo* imaging up to the cellular level, (iii) detailed phenotyping of the (mal)adaptive immune responses, (iv) molecular characterization allowing to generate polygenic risk scores, which, in case of infectious diseases, also encompasses the pathogens’ genetics, as well as (v) drug monitoring.

In this Research Topic, Tran et al. review the currently available patient-reported outcomes (PRO) for chronic inflammatory diseases, their limitations and challenges of addressing these. Especially the longitudinal use of PROs is well-suited to monitor the patient’s perception on the quality of life, disease activity, functional capacity, as well as psychological health. In turn, this enables to capture response (or lack thereof) to treatments in a much more comprehensive and individualized manner (4). Thus, PROs are not only important for clinical management, but also are a cornerstone of more individualized therapies because they directly take individual PROs into account. However, currently clinically implemented PROs only partially reflect actual disease activity. Thus, further development and broader implementation into daily clinical care, of PROs capturing disease activity more precise for individualized therapy guidance is essential for personalized medicine.

Immunophenotyping (Humrich et al.) at a molecular and cellular resolution is a key for classification of patients with chronic inflammatory diseases as it allows to unravel disease pathogenesis and potentially allows to define diagnostic and/or prognostic biomarkers for patient stratification and personalized treatments. Application of unbiased multi-OMICS techniques, such as antigen-specific T cell enrichment (5, 6) or multiplex determination of the activity of 150 kinases (PamGene) (7) are examples of these techniques. The bottleneck of a broader implementation of immunophenotyping are the relative high costs and the integration in a biological and clinical context. For the latter, a close cooperation among clinicians, scientists and systems biologists is essential. Thus, as the tools for phenotyping are available, it will only be a matter of time to explore the full potential of immunophenotyping in inflammation medicine. An example for a biomarker for disease-progression is the presence of myeloid-derived suppressor cells (MDSCs) in hemodialysis patients (Xing et al.): in this patient cohort, persistent high levels of M-MDSCs were associated with higher incidence of stock, heart failure and death. Interestingly, compared to plasma from healthy controls, plasma of hemodialysis patients induced M-MDSCs. This induction of M-MDSCs was sensitive to IL-6 blockade, which may represent a future therapeutic approach for these patients (8). Another example for emerging biomarkers are regulatory autoantibodies to G protein-coupled receptors (GPCR) (Riemekasten et al.), which may reflect the GPCR signature and therefore, the interplay between individual external factors (e.g. microbiome, toxic agents) and internal factors such as the genetic predisposition. Increased or decreased serum concentrations of these autoantibodies lead to clinical disease manifestation (9) and serve as biomarkers for disease progression (10).

Hübenthal et al. highlight the current developments in clinical sequencing and discuss the clinical applicability of polygenic risk scores with regard to chronic inflammatory diseases, such as atopic dermatitis (AD), inflammatory bowel disease (IBD) and coronary artery disease (CAD). Sequencing-based high throughput methods allowed for a (relative) cost-effective sequencing of large patient cohorts. This has and continues to improve our understanding of the genetic background of disease pathogenesis (11–13). In perspective, this data will be the basis for biomedical innovation that potentially allows to for patient stratification at a more individualized level.

Imaging inflammation up to the cellular level is another important pillar for precision medicine. In their overview, Medina et al. review the recent development in imaging of inflammation. Regarding cellular imaging, two-photon microscopy (TPM) for sectioning-free virtual hematoxylin and eosin (H&E) “staining” and optical coherence tomography (OCT) for visualization of cutaneous inflammation. For TPM tissue samples stained with acridine orange (nuclei) and sulforhodamine 101 (counterstain) that leads to an H&E compatible staining. Imaging of unsectioned tissue specimen is then performed using TPM. Ultimately, a digital H&E-equivalent image is generated ready for histological assessment is created from the acquired data. Pending further validation, this workflow of virtual H&E imaging using TPM may represent a faster alternative to conventional histology in the future. The potential of OCT imaging for cellular *in vivo* imaging of the skin has been demonstrated (14). Thus, analysis of cellular morphology combined with dynamic processes of immune cells potentially allows a marker-free “optical biopsy” of skin inflammation using OCT.

Especially in chronic infectious inflammatory diseases, for example tuberculosis, personalized medicine is more and more implemented into clinical care. Biomarker-based treatment decisions, therapeutic drug monitoring and tailored treatments based on the pathogens’ genome are presented in-depth in the review articles by Lange et al. and by Merker et al.

Overall, availability and implementation of those measures will allow individualized treatment decisions. We expect implementation of these in the near future, as respective clinical trials are currently performed. For example, a biomarker discovery trial in prospective cohorts from patients with chronic inflammatory diseases for the definition of disease control, headed by Dr. Schreiber, Dr. Thaci and Dr. Weidinger, or a clinical trial on individualized antibiotic therapy for chronic lung infections headed by Dr. Lange, Dr. Rabe, Dr. Niemann and Dr. Schulenburg, all affiliated with the Cluster of Excellence *Precision Medicine in Chronic Inflammation*.

## Personalized Model Systems

Current pre-clinical model systems usually rely on inbred rodents because of the lower variability of the obtained results (15). Yet, diversity in model systems is a key pre-requisite for basic and translational research in precision medicine. Herein, Tran et al. review the potential use of stem cells and organoid technology in precision medicine in inflammation and highlight the use of organoids from human tissues (16, 17).

## Emergence of Personalized Treatments

Diet is well recognized as an important factor in the pathogenesis of chronic inflammatory diseases, as well as treatment responses (18, 19). Despite this understanding, work demonstrating therapeutic activity of personalized nutrition in chronic inflammation remains scarce. Yet, based on findings in experimental models (20, 21), implementation of precision nutrition in chronic inflammation (Demetrowitsch et al.) will potentially become a therapeutic and/or preventive measure. In line with this notion, insulin is a key factor in host defense as demonstrated by Casagrande et al. Comorbidity of chronic inflammatory diseases is already used for treatment decisions, which is a step towards personalized medicine. For example, use of TNF inhibitors (Zamri and de Vries) in rheumatoid arthritis patients improved periodontal health. As shown by Sang et al., another possibility to personalize medicine could be a targeted delivery of immunomodulatory drugs based on the cell types present in inflammation.

## Perspectives

Identification of unique disease signatures in individual patients is the key to precision medicine in chronic inflammation. This also encompasses the use of personalized model systems for better understanding of disease pathogenesis and selection of treatments. Ultimately, this will lead to the implementation of personalized treatments for patients affected by chronic inflammatory diseases. The future will see that the phenotypes of inflammatory diseases will disintegrate into many rare diseases with targeted therapeutic approaches in small segments of patients.

## Author Contributions

All authors listed have made a substantial, direct, and intellectual contribution to the work and approved it for publication.

## Funding

This work has been financially supported Cluster of Excellence *Precision Medicine in Chronic Inflammation* (EXC 2167) from the Deutsche Forschungsgemeinschaft, the Schleswig-Holstein Excellence-Chair Program from the State of Schleswig Holstein, and Sinergia *Unravel principles of self-organization in injured tissue* (CRSII5_202301/1) from the Swiss National Science Foundation and Skintegrity.ch from the University of Zurich, Switzerland.

## Conflict of Interest

OD has/had consultancy relationship with and/or has received research funding from or has served as a speaker for the following companies in the area of potential treatments for systemic sclerosis and its complications in the last three years: Abbvie, Acceleron, Alcimed, Amgen, AnaMar, Arxx, Baecon, Blade, Bayer, Boehringer Ingelheim, ChemomAb, Corbus, CSL Behring, Galapagos, Glenmark, GSK, Horizon (Curzion), Inventiva, iQvia, Kymera, Lupin, Medac, Medscape, Mitsubishi Tanabe, Novartis, Roche, Roivant, Sanofi, Serodapharm, Topadur and UCB. Patent issued “mir-29 for the treatment of systemic sclerosis” (US8247389, EP2331143). During the last 3 years, RL has received honoraria and/or research grants from the following companies: Admirx, Almirall, Amryth, ArgenX, Biotest, Biogen, Euroimmun, Incyte, Immungenetics, Lilly, Novartis, UCB Pharma, Topadur, True North Therapeutics and Tx Cell. SS has/had consultancy relationship with and has received research funding from the following companies in the area of chronic inflammatory bowel diseases in the last 3 years: Abbvie, Amgen, Arena, Bayer, Biogen, Boehringer Ingelheim, BMS, Celgene, Fresenius Kabi, Galapagos, Gilead, Gossamer, GSK, Hikma, I-Mab, IQvia, Janssen, Lilly, Medscape, MSD, Novartis, Pfizer, Provention Bio, Protagonist, Roche, Sandoz, Shire, Takeda, Theravance, Teva, Tillots, Thermo Fisher.

The remaining authors declare that the research was conducted in the absence of any commercial or financial relationships that could be construed as a potential conflict of interest.

## Publisher’s Note

All claims expressed in this article are solely those of the authors and do not necessarily represent those of their affiliated organizations, or those of the publisher, the editors and the reviewers. Any product that may be evaluated in this article, or claim that may be made by its manufacturer, is not guaranteed or endorsed by the publisher.
